# High level of resistance in the mosquito *Anopheles gambiae* to pyrethroid insecticides and reduced susceptibility to bendiocarb in north-western Tanzania

**DOI:** 10.1186/1475-2875-12-149

**Published:** 2013-05-02

**Authors:** Natacha Protopopoff, Johnson Matowo, Robert Malima, Reginald Kavishe, Robert Kaaya, Alexandra Wright, Philippa A West, Immo Kleinschmidt, William Kisinza, Franklin W Mosha, Mark Rowland

**Affiliations:** 1 Department of Disease Control, London School of Hygiene and Tropical Medicine, Keppel Street, London WC1E 7HT, UK; 2 Kilimanjaro Christian Medical College, Tumaini University, Moshi, Tanzania; 3 National Institute for Medical Research, Amani Medical Research Centre, Muheza, Tanzania; 4 Department of Infectious Disease Epidemiology, London School of Hygiene & Tropical Medicine, Keppel Street, London, UK; 5 MRC Tropical Epidemiology Group, London School of Hygiene & Tropical Medicine, Keppel Street, London, UK

**Keywords:** Insecticide resistance, Knock down mutation, *Anopheles arabiensis*, *Anopheles gambiae*, Tanzania, Pyrethroid, Bendiocarb, Indoor residual spraying, Long-lasting insecticidal net

## Abstract

**Background:**

To control malaria in Tanzania, two primary vector control interventions are being scaled up: long-lasting insecticide-treated nets (LLINs) and indoor residual spraying (IRS). The main threat to effective malaria control is the selection of insecticide resistance. While resistance to pyrethroids, the primary insecticide used for LLINs and IRS, has been reported among mosquito vectors in only a few sites in Tanzania, neighbouring East African countries are recording increasing levels of resistance. To monitor the rapidly evolving situation, the resistance status of the malaria vector *Anopheles gambiae* s.l to different insecticides and the prevalence of the *kdr* resistance allele involved in pyrethroid resistance were investigated in north-western Tanzania, an area that has been subject to several rounds of pyrethroid IRS since 2006.

**Methods:**

Household collections of anopheline mosquitoes were exposed to diagnostic dosages of pyrethroid, DDT, and bendiocarb using WHO resistance test kits. The relative proportions of *An. gambiae* s.s and *Anopheles arabiensis* were also investigated among mosquitoes sampled using indoor CDC light traps. Anophelines were identified to species and the *kdr* mutation was detected using real time PCR TaqMan assays.

**Results:**

From the light trap collections 80% of *An. gambiae* s.l were identified as *An. gambiae* s.s and 20% as *An. arabiensis*. There was cross-resistance between pyrethroids and DDT with mortality no higher than 40% reported in any of the resistance tests. The *kdr*-eastern variant was present in homozygous form in 97% of *An. gambiae* s.s but was absent in *An. arabiensis*. *Anopheles gambiae* s.s showed reduced susceptibility to the carbamate insecticide, bendiocarb, the proportion surviving WHO tests ranging from 0% to 30% depending on season and location.

**Conclusion:**

*Anopheles gambiae* s.s has developed phenotypic resistance to pyrethroids and DDT and *kdr* frequency has almost reached fixation. Unlike in coastal Tanzania, where the ratio of *An. gambiae* s.s to *An. arabiensis* has decreased in response to vector control, *An. gambiae* s.s persists at high frequency in north-western Tanzania, probably due to selection of pyrethroid resistance, and this trend is likely to arise in other areas as resistance spreads or is subject to local selection from IRS or LLINs.

## Background

Strong commitment from international agencies and home governments to reduce the burden of malaria in sub-Saharan Africa has led to a major scale-up of vector control measures and increased access to effective anti-malarial treatment, and it is reported that malaria is on the wane in several African countries [1]. However a major menace is threatening the present achievements. Resistance to pyrethroid insecticide is spreading rapidly across Africa and could reduce the impact of our two most successful malaria prevention interventions - indoor residual spraying (IRS) and long-lasting insecticidal nets (LLINs) [2-4]. The two main mechanisms responsible for pyrethroid resistance are target site insensitivity, known as knock down resistance *kdr,* and metabolic resistance due to elevated levels of detoxifying enzymes [2]. *Kdr* is caused by mutations to the sodium channel, a leucine to phenylalanine change first observed in West Africa [5] and a leucine to serine mutation observed in East Africa [6]. Recently a new mutation in the sodium channel conferring additional resistance to DDT and permethrin as been reported associated with the *kdr*-west mutation [7].

To reduce the malaria burden in Tanzania, the National Malaria Control Programme (NMCP) is increasing the coverage of LLINs and IRS. Over the last 10 years LLINs have been distributed initially by targeting the most vulnerable groups, pregnant women and children aged under five, through discounted vouchers issued at antenatal clinics [8], and then by a national, free LLIN distribution campaign in 2010 [9] which was extended to the general population though a universal coverage LLIN distribution campaign in 2011. IRS with pyrethroid was initiated in Kagera region, situated on the western shore of Lake Victoria, in 2006 with support from the President’s Malaria Initiative and extended to all Lake Zone in 2011.

Previous surveys conducted in Tanzania showed little or no resistance to DDT and pyrethroids in the *Anopheles gambiae* s.l population [10-12]. A study carried out in 2009/2010 showed no resistance to deltamethrin and DDT in Muleba [10], north-western district of Tanzania, where the present study is conducted. In this paper, the insecticide resistance status of the malaria vectors *An. gambiae* s.s and *Anopheles arabiensis* to insecticides in use for IRS (lambdacyhalothrin and bendiocarb) and LLIN (permethrin and deltamethrin) is presented. The prevalence of the *kdr* mutation was also investigated.

## Methods

### Study area

**Figure 1:**
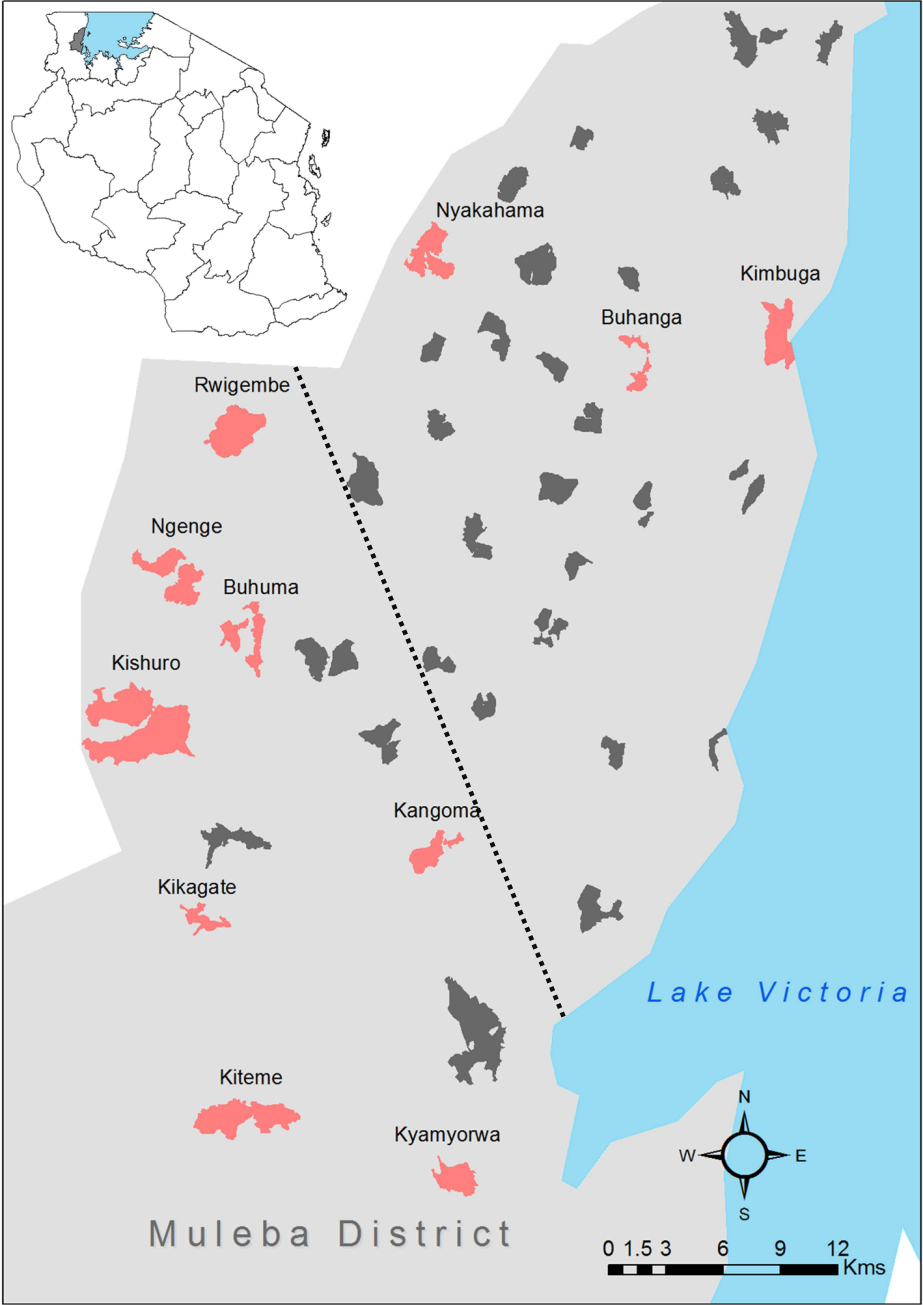
**Map of Tanzania showing the study area (top left).** Map showing villages where sampling was conducted. Sites, for morning resting collection to perform WHO resistance test and light trap collection, are shown in red. Sites where only light trap collection was done are in dark grey. The dotted line delimited the high density of *Anopheles* area in the south west from the low *Anopheles* density area in the north east.

The study was carried out in Muleba district (1°45’S, 31^o^40’E) in Kagera region of north-western Tanzania on the western shore of Lake Victoria (Figure 1) as a component of a cluster randomized trial investigating the combined use of IRS and LLINS for malaria prevention (clinical trials identifier # NCT01697852).

The study area is situated at 1,100-1,600 m above sea level. Since 2006 five rounds of IRS with the pyrethroid lambdacyhalothrin (ICON 10CS, Syngenta, Basel, Switzerland) were conducted in Muleba district. Since the present study was completed, two rounds of IRS have been carried out with the carbamate bendiocarb (FICAM 80% Wettable Power, Bayer) in December 2011 and May 2012. Net coverage has increased since 2005 through net distribution campaigns initially targeting pregnant women and children aged under five in 2009 (63% of households provided with nets) and then targeting the entire population at risk in 2011 (91% household ownership) [13].

### Mosquito collection

Monthly rounds of mosquito collections were carried out in 40 villages (Figure 1) from April to December 2011 using CDC light traps. During each monthly round of mosquito collecting, light trapping was conducted for one night in eight households selected at random from each village. Light traps were installed at the foot of human occupied beds covered with a treated or untreated bed net. Each of the villages had been subjected to an earlier round of pyrethroid IRS in January-February 2011.

Adult *Anopheles* were collected for WHO insecticide susceptibility testing in April-June and November-December 2011 in 11 villages by means of morning indoor resting catches using suction tubes. The collections were identified to species using a simplified morphological key adapted from Gillies and Coetzee [14] and stored individually for molecular identification and detection of *kdr* variants.

### WHO insecticide resistance tests

The collections of adult *An. gambiae* s.l were tested in standard WHO resistance test kit using discriminating dosages of the pyrethroids lambdacyhalothrin (0.05%), permethrin (0.75%) and deltamethrin (0.05%) and with DDT (4%) and bendiocarb (0.1%) [15]. WHO test and control papers were supplied by the WHO Collaborating Centre at Universiti Sains Malaysia, Penang, Malaysia. Test papers were used no more than five times before being replaced. Anopheline mosquitoes were exposed to the insecticides for one hour and mortality was scored after a 24-hour holding period [15] during which the *Anopheles* had access to sugar solution. Tests were excluded if control mortality exceeded 5%. Physiological/gonotrophic status was recorded. Tests with the same impregnated papers were performed against the *An. gambiae* s.s Kisumu susceptible strain as a check on the quality of the test papers.

### Mosquito species identification and genotyping for *kdr* mutations

Genomic DNA was extracted from body parts (leg, antenna or wings) of *An. gambiae* s.l and was stored at −20°C until use. Real time PCR using TaqMan assays was used to distinguish between the two sibling species *An. gambiae* s.s and *An .arabiensis*[16] and to distinguish between *kdr* east and *kdr* west genotypes (*kdr-w* or *kdr-e*) [17]. Genotyping results were analysed using MXPro software (Agilent technologies, Stratagene, USA).

### Data analysis

Percentage mortality and 95% confidence interval in WHO susceptibility tests was calculated by the binomial exact method using Stata 11 (Stata-Corporation, USA). Regardless of the total number of *An. gambiae* s.l collected in light traps from a given cluster, the maximum subsample tested was limited to 50 individuals. The proportions of *An. gambiae* and *An. arabiensis* were determined in each subsample and weighted by the inverse of the sampling fraction (ie, subsample/total collected) to represent the relative proportion in the total population. Analysis of the difference in proportion of *An. arabiensis* and fed *Anopheles* between tests and months was done using logistic regression. *Kdr* genotype frequencies among dead and alive *An. gambiae* s.l in WHO tests and light trap collections across different rounds and clusters were compared using the software Genepop (version 4.0) [18]. The study map was designed using ArcGIS 10 (ESRI Japan Corp, Tokyo, Japan.).

### Ethics statement

The trial was approved by the ethics review committees of the Kilimanjaro Christian Medical College, the National Institute for Medical Research Tanzania and the London School of Hygiene and Tropical Medicine (application no. 5814). Written informed consent was obtained from adults of each house where collections were made.

## Results

### Light trap collection

Seven rounds of light trap collection were completed in the 40 villages between April and December 2011 and a total of 5,762 *An. gambiae* s.l females were caught. The majority of mosquitoes (92%) were collected from 13 villages situated in the south-west of the district (Figure 1). A subsample of 2,346 was identified to species. The weighted proportion showed a ratio of 80% (95% CI: 75%-84%) *An. gambiae* s.s to 20% (95% CI: 16%-25%) *An.arabiensis.*

### WHO resistance tests

*Anopheles gambiae* s.s accounted for 96.0% and *An. arabiensis* for 3.7% of the *An. gambiae* s.l tested for resistance (N = 901). *Anopheles arabiensis* was detected only in four sites, and made up 7.2% of *An. gambiae* s.l in Kishuro and 3.4% in Kikagate in surveys done in May, and 2.6% in Kyamorwa and 18.2% in Kiteme in the surveys done in November. All 100% *An. arabiensis* were killed in tests with, lambdacyhalothrin (n = 11), deltamethrin (n = 2), DDT (n = 10) and bendiocarb (n = 2), and 50% (4/8) in tests with the pyrethroid permethrin.

*Anopheles gambiae* s.s mortality to lambdacyhalothin 0.05% test papers ranged from 8% to 40% (Table 1) across the 11 villages sampled, indicating a high frequency of resistance in the area and some variation in resistance frequency by cluster. Mosquitoes tested in two villages, Kyarmorwa and Kikagate, showed a reduction in percentage mortality between May and November 2011 (from 34% to 8% in Kyarmorwa and from 40% to 26% in Kikagate). The resistance to lambdacyhalothrin extended to permethrin and deltamethrin (Table 2) and also showed cross-resistance to DDT with mortality ranging from 13% to 40% between villages. Similar temporal trends between surveys were observed to DDT as to lambdacyhothrin, indicating cross-resistance between DDT and pyrethroids.

**Table 1:** **Mortality rates (95% confidence interval) and blood-feeding status of *Anopheles gambiae *s.l from various localities exposed to lambdacyhalothrin 0.05% in WHO resistance tests**

**Date of testing**	**Village**	**Total tested**	**Replicate no.**	** % mortality (95%CI)**		**% blood-fed *Anopheles***	***Kdr *allele frequency**
May 2011	Kikagate	35	2	34%	(19–52)	100%	95%
	Kyamyorwa	230	12	40%^3^	(33–46)	70%	100%
Nov 2011	Kishuro	40	2	13%	(4–27)	24%	99%
	Ngenge	52	3	25%	(14–39)	59%	99%
	Rwigembe	42	2	31%	(18–47)	84%	100%
	Buhuma	30	2	17%	(6–35)	78%	100%
	Kangoma	105	6	8%^1^	(3–14)	40%	96%
	Kikagate	142	7	8%	(4–14)	74%	98%
	Kiteme	104	6	31%^2^	(22–41)	92%	99%
	Kyamyorwa	149	7	26%^3^	(19–34)	75%	98%
	Nyakahama	13	1	38%	(14–68)	77%	100%
	Buhanga	36	2	22%^3^	(10–39)	86%	98%
	Kimbuga	21	2	0%	(0–16)	43%	94%
	Kisumu strain	100	4	100%	(96–100)		

The allelic frequency of the *kdr* east mutation among *An. gambiae* s.s is reported separately for each village.

^1^Control mortality was 2%, ^2^3% and ^3^4% in the test indicated with a subscript and was 0% in all the other tests.

**Table 2:** **Mortality rates of the *Anopheles gambiae *s.l field populations from various localities exposed to deltamethrin, permethrin, DDT and bendiocarb**

**Insecticide**	**Date**	**Village**	**Total tested**	**No. of replicates**	** % mortality (95%CI)**		**% blood-fed *Anopheles***
Bendiocarb (0.1%)	May 2011	Kyamyorwa	112	6	100%	(97–100)	100%
	Nov 2011	Kyamyorwa	106	7	84%^1^	(76–90)	78%
		Kangoma	54	3	70%^3^	(56–82)	86%
		Kikagate	100	5	86%	(78–92)	65%
		Kiteme	84	5	90%^2^	(82–96)	96%
		Kisumu strain	61	4	97%	(89–100)	
DDT (4%)	May 2011	Kyamyorwa	98	5	37%	(27–47)	100%
	Nov 2011	Kyamyorwa	99	5	13%^1^	(7–21)	88%
		Rwigembe	8	1	13%	(0–53)	36%
		Buhuma	12	1	17%	(2–48)	58%
		Kikagate	20	1	35%	(15–59)	55%
		Kiteme	85	4	40%^2^	(30–51)	100%
		Kisumu strain	100	4	100%	(96–100)	
Deltamethrin (0.05%)	May 2011	Kishuro	20	1	70%	(46–88)	100%
		Kyamyorwa	106	5	28%^3^	(20–38)	100%
Permethrin (0.75%)	Nov 2011	Kiteme	98	5	11%	(6–19)	98%

Proportion of blood-fed *Anopheles* at the time of the testing is reported.

^1^Control mortality was 2%, ^2^3% and ^3^4% in the test indicated with a subscript and was 0% in all the other test.

*Anopheles gambiae* s.s showed reduced susceptibility to the carbamate bendiocarb in WHO resistance tests. Percentage mortality in the village Kyarmorwa was 100% in May 2011 and 84% in November 2011. Tests, done in other villages in November, produced mortality rates ranging from 70% to 90%. Checks done on the *An. gambiae* Kisumu susceptible strain produced 100% mortality on DDT and lambdacyhalothrin and 97% mortality with the bendiocarb test papers.

Of the *An. gambiae* collected for testing, 79% were blood-fed, 14% were unfed and the remaining were gravid or semi-gravid (Table 1). There were variations in blood-feeding rates between the tests, however no differences in frequency of fed mosquitoes were observed in alive and dead mosquitoes exposed to lambdacyhalothrin (p = 0.15).

### *Kdr* mutation

Of the 2,049 *An. gambiae* s.s collected by light trap and tested for the *kdr* east allele, 96.8% (n = 1,983) were homozygous for *kdr*, 3% (n = 62) were heterozygous and only 0.2% (n = 4) were homozygous for the susceptible type. All *An. arabiensis* tested (N = 297) were homozygous for susceptible type. There was no significant difference in genotype frequency in *An. gambiae* s.s between collection rounds (Chi2 = 1.2, df = 2, P = 0.55) or villages (Chi2 = 2.5, df = 2, P = 0.29).

*Kdr* genotype frequencies in the *An. gambiae* s.s collected resting in houses (later exposed to WHO resistance tests) were similar to the frequencies in the light trap collections. Of the 772 *An. gambiae* s.s tested 96.9% (747) were homozygous for *kdr*, 3.0% (24) were heterozygous and only one was wild type. None of the *An. arabiensis* tested (n = 31) carried the *kdr* east mutation. No *kdr* west mutation was found in any of the 176 *An. gambiae* s.s or *An. arabiensis* tested.

Allelic frequency was compared between *An. gambiae* s.s surviving or dying in the pyrethroid resistance tests. Because the *kdr* frequency was almost fixed (98%) there was no association between *kdr*-e allele frequency and the phenotypic resistance in WHO tests (lambdacyhalothin, permethrin, deltamethrin p-value = 1.0, DDT p-value = 0.59).

## Discussion

*Anopheles gambiae* s.s resistance to pyrethroids and DDT was widespread throughout the study area of Muleba district in north-western Tanzania. The frequency of the *kdr* east approached fixation in the *An. gambiae* s.s population but was absent in *An. arabiensis*. Emerging resistance to bendiocarb was observed for the first time.

There was considerable variation in the density of mosquitoes between clusters. Greater densities of mosquitoes were to be found in the south-west. It was important to test samples of *Anopheles* from the north-east area to investigate resistance as a possible cause of the heterogeneity in *Anopheles* density between the two areas. While larger samples from the north-east were desirable, this was not possible with the sampling plan and resources available. The level of mortality recorded in the resistance tests was low and never exceed 40% in any of the clusters (and always read against a control) regardless the sample size tested and hence considerable confidence can be placed in the overall trend in resistance.

The samples for testing were deliberately chosen from adult collections to represent natural age-structured populations. These would be a mix of young and old mosquitoes, and because the level of resistance often decreases in ageing mosquitoes [19,20] the proportion surviving in the tests would have been higher had, for example, F1 adults reared from larval collections been chosen for testing instead. The disadvantage of using larval collections is the limited gene pool of the collected samples, and the possible bias in resistance frequency which is much less likely to occur with adult collections. While it is possible that the adult collection was under selection from decaying pyrethroid residues in houses, the resistance frequency would still be representative of the population.

In the resistance tests with lambdacyhalothrin on mosquitoes that were identified by PCR, 23% of *An. gambiae* s.s and 100% of *An. arabiensis* were killed overall. This is the first time that high-level pyrethroid resistance and a high frequency of the *kdr* east mutation is reported in Tanzania. A national resistance survey conducted in 2009/2010 in 12 sentinel sites indicated that resistance is starting to be detected in other parts Tanzania but not at the levels found in Muleba district of Kagera region where IRS with lambdacyhalothrin has been intensively applied between 2006 and 2011 [10]. In the Lower Moshi agricultural zone of Kilimanjaro region, for example, *An. arabiensis* is the predominant species and is resistant to permethrin (13%), attributed to elevated levels of mixed function oxidases rather than *kdr* as the species is still fully susceptible to DDT [12], *kdr* east has not been recorded [21] and *kdr* west is present at very low frequency [22]. Kagera region, however, borders on neighbouring countries and resistance findings in the present study site is more similar of Uganda [23], Burundi [24] and Kenya [25] where phenotypic resistance to pyrethroids and DDT is high and *kdr* east allele reported at a high frequency.

The high prevalence of pyrethroid resistance and high frequency of *kdr* might be a response to selection by recurrent IRS with lambdacyhalothin since it is found nowhere else at this frequency and no other region of Tanzania has been under such intense selection pressure from pyrethroid IRS since 2006. However, it is possible that kdr present in *An. gambiae* in a neighbouring area spread to Muleba by migration. It is not clear whether the resistance to bendiocarb is independent of the resistance to pyrethroids or if there is a common mechanism [26] arising from pyrethroid selection since bendiocarb resistance was detected before it was used as IRS.

The implications of the pyrethroid resistance on the operational impact of vector control measures, particularly LLINs, are currently uncertain. In West Africa, in areas of high resistance, LLINs show reduced effectiveness against vector populations [27,28]. Control failure attributed to insecticide resistance has been observed after IRS campaigns in South Africa [2,29] and the island of Bioko [4]. While historically in some West African countries ITN/LLINs provide some protection against *kdr* resistant *Anopheles* populations [30-32], that situation appears to be changing with selection of additional metabolic mechanisms [27,28]. In Burundi, the high frequency of *kdr* did not lead to a loss of efficacy of IRS [24,33]. In western Kenya where *kdr* in *An. gambiae* s.s was reaching fixation, a species shift occurred towards the more zoophilic sibling species *An. arabiensis*[25] despite *kdr* being selected in *An. gambiae* s.s This has been attributed to the increased use of LLIN and their continued effectiveness against *An. gambiae* s.s [34]. Unlike western Kenya and the coast of Tanzania [35], in Muleba, *An. gambiae* s.s is still the predominant species of the *gambiae* complex despite several rounds of pyrethroid IRS and increased use of LLINs. The possible selection of supplementary resistance mechanisms in *An.gambiae* s.s based on enhanced metabolism may explain the high prevalence of resistance and persistence of *An.gambiae* s.s in our study area, as it has been reported in a different area of Kenya [36], but this has yet to be confirmed.

## Conclusions

*Anopheles gambiae* s.s has developed high resistance to pyrethroids and DDT and *kdr* frequency has almost reached fixation in north-western Tanzania. Bendiocarb resistance is also emerging in this vector. Further investigation will be needed to understand the mechanism underlying the phenotypic resistance to pyrethroids in the area and evaluate the potential operational impact of insecticide resistance in order to guide the selection of suitable insecticides and vector control interventions. Meanwhile resistance management strategies [37] should be considered and implemented to delay the expansion of insecticide resistance to other areas of Tanzania.

## Competing interests

The authors declare that they have no competing interests.

## Authors’ contributions

NP was involved in the study design, supervised the implementation of the study and data collection, analysed data, drafted and revised the manuscript. JM was involved in data collection, helped to analyse the data, draft and revised the manuscript. RM was involved in the study design, implementation and supervision of the data collection and revised the manuscript. ReK, RoK and AW performed the real time PCR testing and revised the manuscript. PW was involved in the study design, supported the field work and revised the manuscript. IK, WK and FM were involved in the overall trial design, helped to draft and revised the manuscript. MR was involved in study design, interpretation of the data and revisions of the manuscript. All authors have read and approved the final version of the manuscript.
